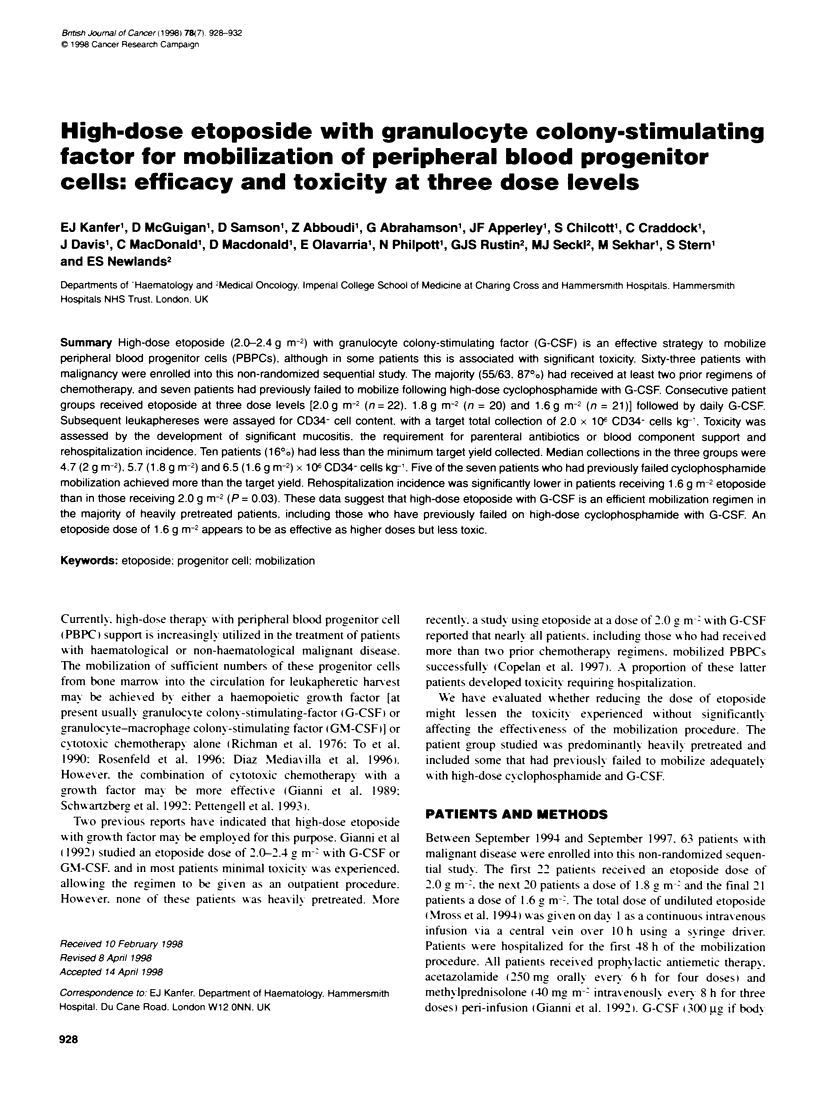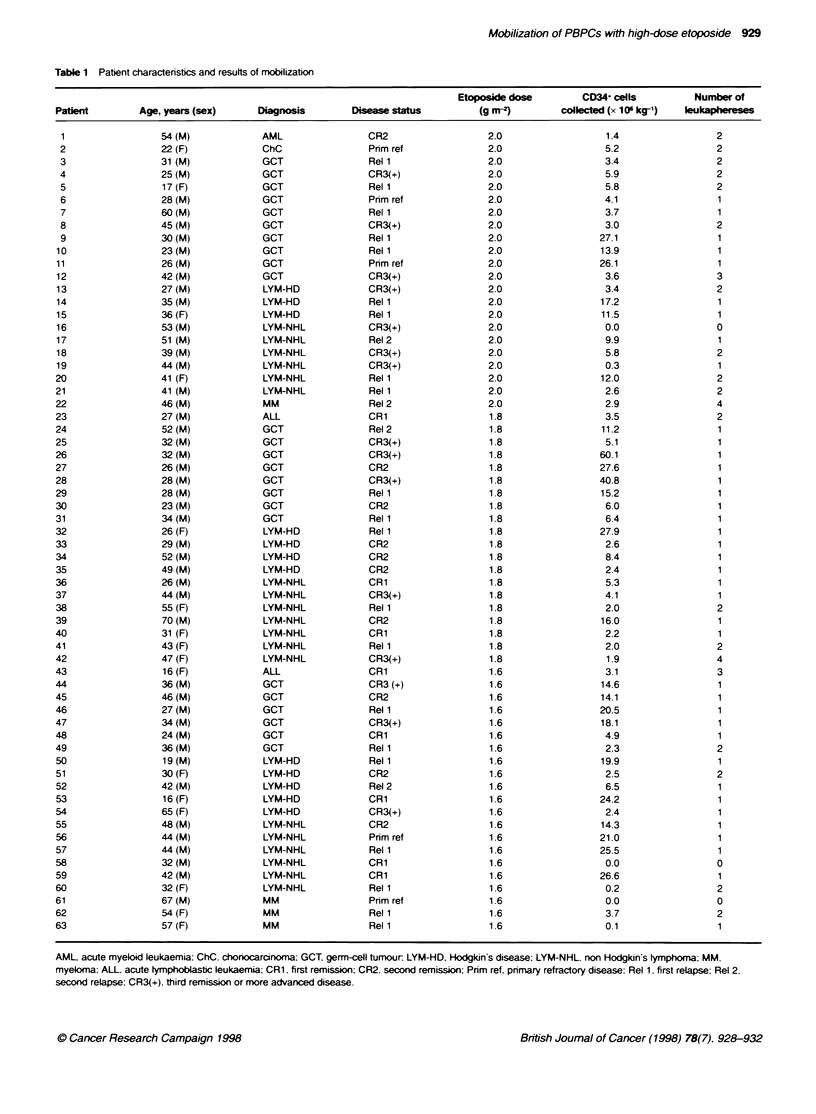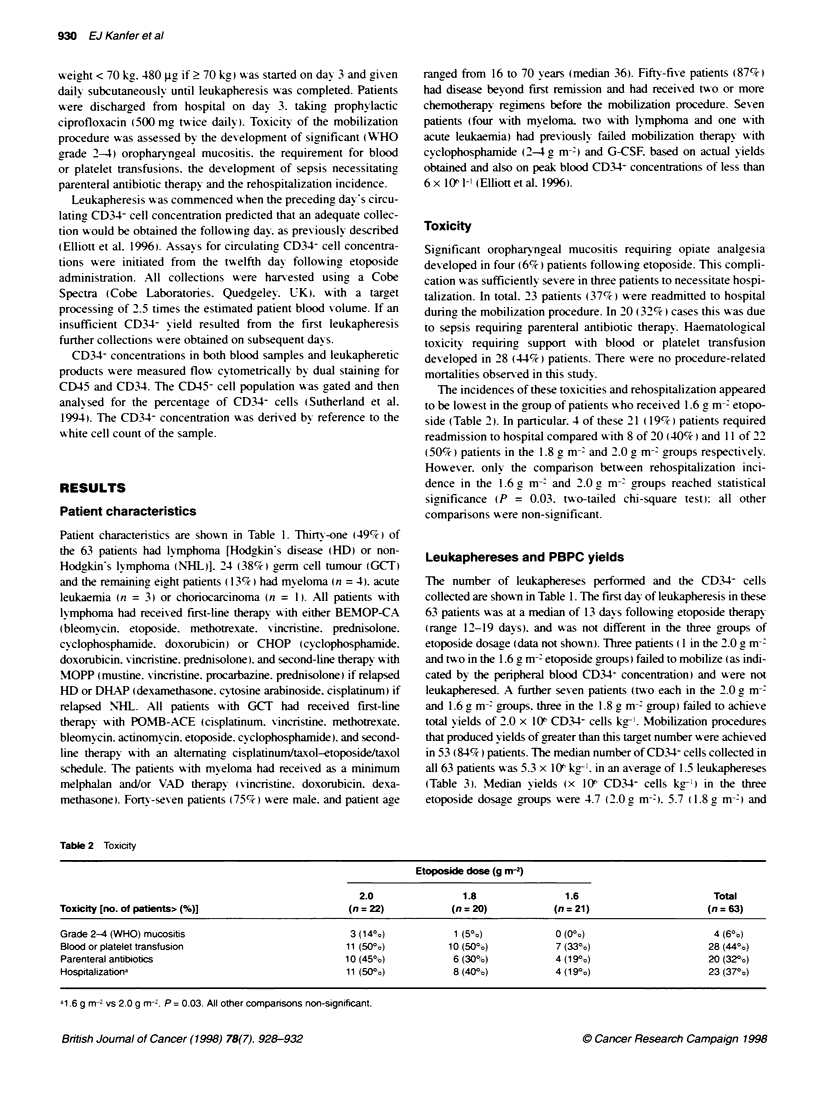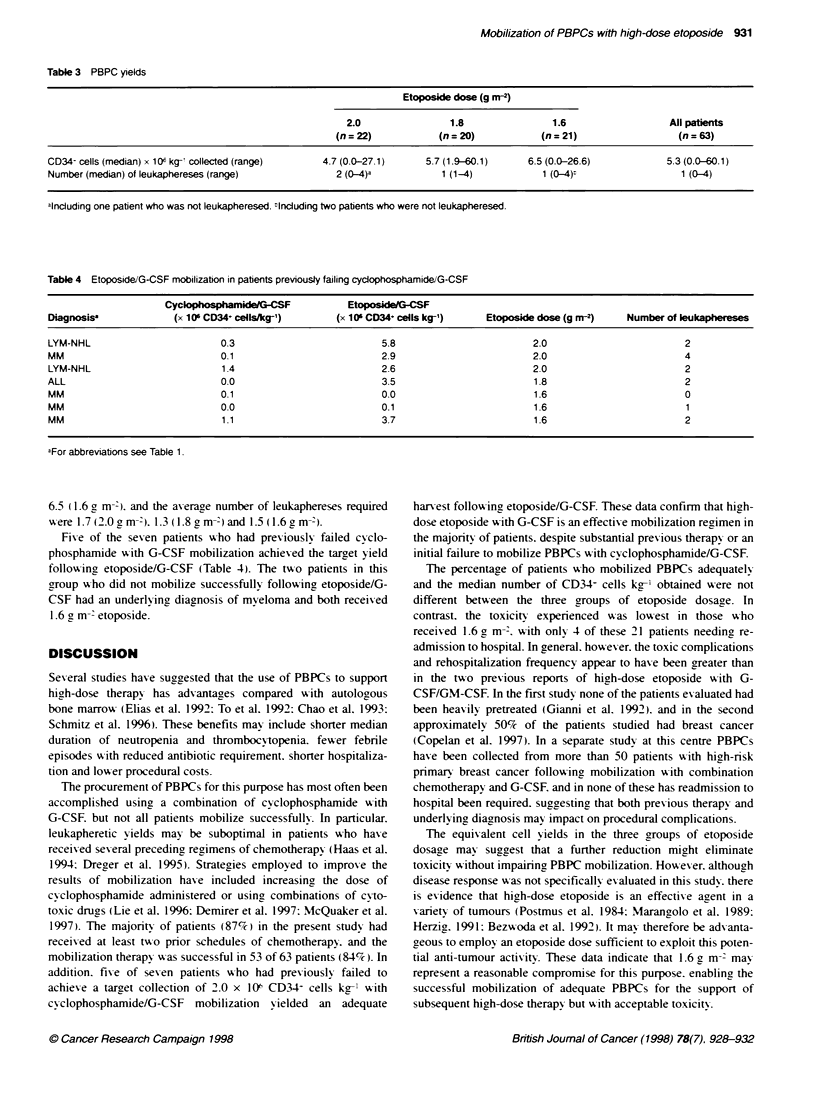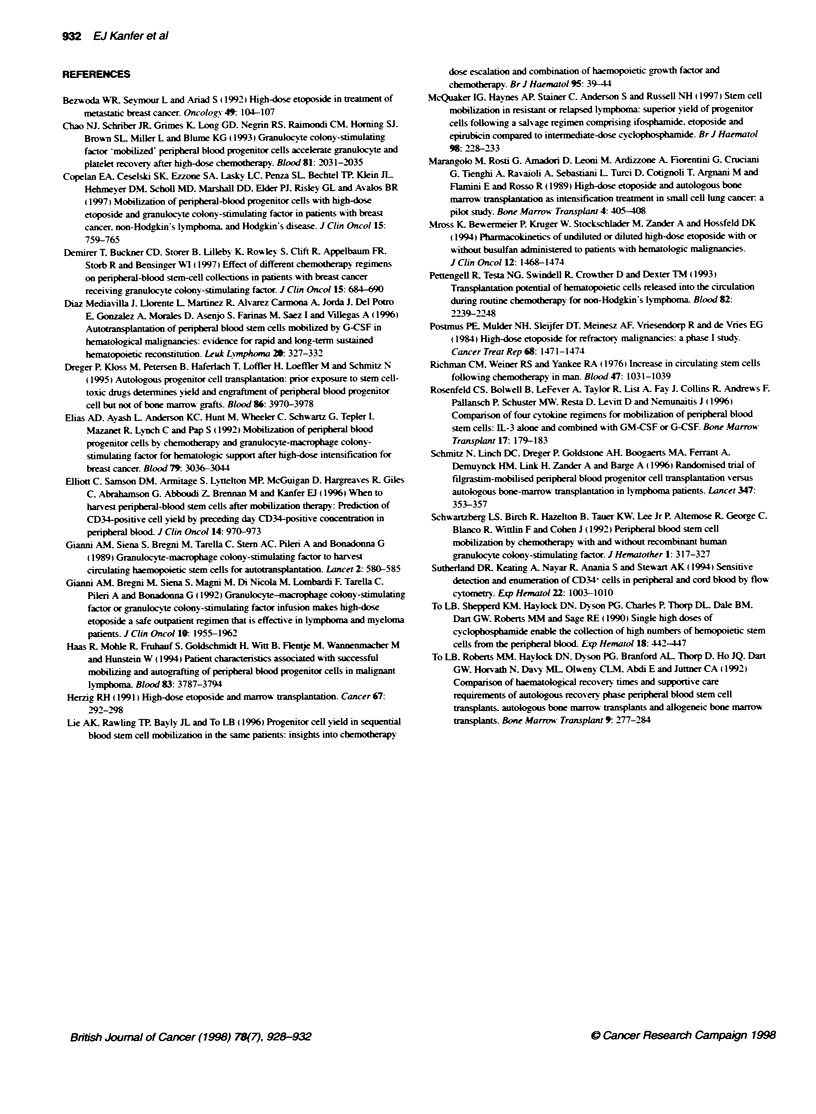# High-dose etoposide with granulocyte colony-stimulating factor for mobilization of peripheral blood progenitor cells: efficacy and toxicity at three dose levels.

**DOI:** 10.1038/bjc.1998.603

**Published:** 1998-10

**Authors:** E. J. Kanfer, D. McGuigan, D. Samson, Z. Abboudi, G. Abrahamson, J. F. Apperley, S. Chilcott, C. Craddock, J. Davis, C. MacDonald, D. Macdonald, E. Olavarria, N. Philpott, G. J. Rustin, M. J. Seckl, M. Sekhar, S. Stern, E. S. Newlands

**Affiliations:** Department of Haematology, Imperial College School of Medicine at Charing Cross Hospital, Hammersmith Hospitals NHS Trust, London, UK.

## Abstract

High-dose etoposide (2.0-2.4 g m(-2)) with granulocyte colony-stimulating factor (G-CSF) is an effective strategy to mobilize peripheral blood progenitor cells (PBPCs), although in some patients this is associated with significant toxicity. Sixty-three patients with malignancy were enrolled into this non-randomized sequential study. The majority (55/63, 87%) had received at least two prior regimens of chemotherapy, and seven patients had previously failed to mobilize following high-dose cyclophosphamide with G-CSF. Consecutive patient groups received etoposide at three dose levels [2.0 g m(-2) (n = 22), 1.8 g m(-2) (n = 20) and 1.6 g m(-2) (n = 21)] followed by daily G-CSF. Subsequent leukaphereses were assayed for CD34+ cell content, with a target total collection of 2.0 x 10(6) CD34+ cells kg(-1). Toxicity was assessed by the development of significant mucositis, the requirement for parenteral antibiotics or blood component support and rehospitalization incidence. Ten patients (16%) had less than the minimum target yield collected. Median collections in the three groups were 4.7 (2 g m(-2)), 5.7 (1.8 g m(-2)) and 6.5 (1.6 g m(-2)) x 10(6) CD34+ cells kg(-1). Five of the seven patients who had previously failed cyclophosphamide mobilization achieved more than the target yield. Rehospitalization incidence was significantly lower in patients receiving 1.6 g m(-2) etoposide than in those receiving 2.0 g m(-2) (P = 0.03). These data suggest that high-dose etoposide with G-CSF is an efficient mobilization regimen in the majority of heavily pretreated patients, including those who have previously failed on high-dose cyclophosphamide with G-CSF. An etoposide dose of 1.6 g m(-2) appears to be as effective as higher doses but less toxic.


British Journal of Cancer (1998) 78(7), 928–932
© 1998 Cancer Research Campaign

### High-dose etoposide with granulocyte colony-stimulating factor for mobilization of peripheral blood progenitor cells: efficacy and toxicity at three dose levels

**EJ Kanfer[1], D McGuigan[1], D Samson[1], Z Abboudi[1], G Abrahamson[1], JF Apperley[1], S Chilcott[1], C Craddock[1], J Davis[1], C MacDonald[1], D Macdonald[1], E Olavarria[1], N Philpott[1], GJS Rustin[2], MJ Seckl[2], M Sekhar[1], S Stern[1] and ES Newlands[2]**

Departments of [1]Haematology and [2]Medical Oncology, Imperial College School of Medicine at Charing Cross and Hammersmith Hospitals, Hammersmith Hospitals NHS Trust, London, UK

**Summary** High-dose etoposide (2.0–2.4 g $m^{-2}$) with granulocyte colony-stimulating factor (G-CSF) is an effective strategy to mobilize peripheral blood progenitor cells (PBPCs), although in some patients this is associated with significant toxicity. Sixty-three patients with malignancy were enrolled into this non-randomized sequential study. The majority (55/63, 87%) had received at least two prior regimens of chemotherapy, and seven patients had previously failed to mobilize following high-dose cyclophosphamide with G-CSF. Consecutive patient groups received etoposide at three dose levels [2.0 g $m^{-2}$ ($n = 22$), 1.8 g $m^{-2}$ ($n = 20$) and 1.6 g $m^{-2}$ ($n = 21$)] followed by daily G-CSF. Subsequent leukaphereses were assayed for $CD34^+$ cell content, with a target total collection of 2.0 × $10^6$ $CD34^+$ cells $kg^{-1}$. Toxicity was assessed by the development of significant mucositis, the requirement for parenteral antibiotics or blood component support and rehospitalization incidence. Ten patients (16%) had less than the minimum target yield collected. Median collections in the three groups were 4.7 (2 g $m^{-2}$), 5.7 (1.8 g $m^{-2}$) and 6.5 (1.6 g $m^{-2}$) × $10^6$ $CD34^+$ cells $kg^{-1}$. Five of the seven patients who had previously failed cyclophosphamide mobilization achieved more than the target yield. Rehospitalization incidence was significantly lower in patients receiving 1.6 g $m^{-2}$ etoposide than in those receiving 2.0 g $m^{-2}$ ($P = 0.03$). These data suggest that high-dose etoposide with G-CSF is an efficient mobilization regimen in the majority of heavily pretreated patients, including those who have previously failed on high-dose cyclophosphamide with G-CSF. An etoposide dose of 1.6 g $m^{-2}$ appears to be as effective as higher doses but less toxic.

**Keywords:** etoposide; progenitor cell; mobilization

Currently, high-dose therapy with peripheral blood progenitor cell (PBPC) support is increasingly utilized in the treatment of patients with haematological or non-haematological malignant disease. The mobilization of sufficient numbers of these progenitor cells from bone marrow into the circulation for leukapheretic harvest may be achieved by either a haemopoietic growth factor [at present usually granulocyte colony-stimulating-factor (G-CSF) or granulocyte–macrophage colony-stimulating factor (GM-CSF)] or cytotoxic chemotherapy alone (Richman et al, 1976; To et al, 1990; Rosenfeld et al, 1996; Diaz Mediavilla et al, 1996). However, the combination of cytotoxic chemotherapy with a growth factor may be more effective (Gianni et al, 1989; Schwartzberg et al, 1992; Pettengell et al, 1993).

Two previous reports have indicated that high-dose etoposide with growth factor may be employed for this purpose. Gianni et al (1992) studied an etoposide dose of 2.0–2.4 g $m^{-2}$ with G-CSF or GM-CSF, and in most patients minimal toxicity was experienced, allowing the regimen to be given as an outpatient procedure. However, none of these patients was heavily pretreated. More recently, a study using etoposide at a dose of 2.0 g $m^{-2}$ with G-CSF reported that nearly all patients, including those who had received more than two prior chemotherapy regimens, mobilized PBPCs successfully (Copelan et al, 1997). A proportion of these latter patients developed toxicity requiring hospitalization.

We have evaluated whether reducing the dose of etoposide might lessen the toxicity experienced without significantly affecting the effectiveness of the mobilization procedure. The patient group studied was predominantly heavily pretreated and included some that had previously failed to mobilize adequately with high-dose cyclophosphamide and G-CSF.

### PATIENTS AND METHODS

Between September 1994 and September 1997, 63 patients with malignant disease were enrolled into this non-randomized sequential study. The first 22 patients received an etoposide dose of 2.0 g $m^{-2}$, the next 20 patients a dose of 1.8 g $m^{-2}$ and the final 21 patients a dose of 1.6 g $m^{-2}$. The total dose of undiluted etoposide (Mross et al, 1994) was given on day 1 as a continuous intravenous infusion via a central vein over 10 h using a syringe driver. Patients were hospitalized for the first 48 h of the mobilization procedure. All patients received prophylactic antiemetic therapy, acetazolamide (250 mg orally every 6 h for four doses) and methylprednisolone (40 mg $m^{-2}$ intravenously every 8 h for three doses) peri-infusion (Gianni et al, 1992). G-CSF (300 μg if body

Received 10 February 1998
Revised 8 April 1998
Accepted 14 April 1998

Correspondence to: EJ Kanfer, Department of Haematology, Hammersmith Hospital, Du Cane Road, London W12 0NN, UK

**Table 1** Patient characteristics and results of mobilization

| Patient | Age, years (sex) | Diagnosis | Disease status | Etoposide dose (g $m^{-2}$) | $CD34^+$ cells collected ($\times 10^6$ $kg^{-1}$) | Number of leukaphereses |
|---|---|---|---|---|---|---|
| 1 | 54 (M) | AML | CR2 | 2.0 | 1.4 | 2 |
| 2 | 22 (F) | ChC | Prim ref | 2.0 | 5.2 | 2 |
| 3 | 31 (M) | GCT | Rel 1 | 2.0 | 3.4 | 2 |
| 4 | 25 (M) | GCT | CR3(+) | 2.0 | 5.9 | 2 |
| 5 | 17 (F) | GCT | Rel 1 | 2.0 | 5.8 | 2 |
| 6 | 28 (M) | GCT | Prim ref | 2.0 | 4.1 | 1 |
| 7 | 60 (M) | GCT | Rel 1 | 2.0 | 3.7 | 1 |
| 8 | 45 (M) | GCT | CR3(+) | 2.0 | 3.0 | 2 |
| 9 | 30 (M) | GCT | Rel 1 | 2.0 | 27.1 | 1 |
| 10 | 23 (M) | GCT | Rel 1 | 2.0 | 13.9 | 1 |
| 11 | 26 (M) | GCT | Prim ref | 2.0 | 26.1 | 1 |
| 12 | 42 (M) | GCT | CR3(+) | 2.0 | 3.6 | 3 |
| 13 | 27 (M) | LYM-HD | CR3(+) | 2.0 | 3.4 | 2 |
| 14 | 35 (M) | LYM-HD | Rel 1 | 2.0 | 17.2 | 1 |
| 15 | 36 (F) | LYM-HD | Rel 1 | 2.0 | 11.5 | 1 |
| 16 | 53 (M) | LYM-NHL | CR3(+) | 2.0 | 0.0 | 0 |
| 17 | 51 (M) | LYM-NHL | Rel 2 | 2.0 | 9.9 | 1 |
| 18 | 39 (M) | LYM-NHL | CR3(+) | 2.0 | 5.8 | 2 |
| 19 | 44 (M) | LYM-NHL | CR3(+) | 2.0 | 0.3 | 1 |
| 20 | 41 (F) | LYM-NHL | Rel 1 | 2.0 | 12.0 | 2 |
| 21 | 41 (M) | LYM-NHL | Rel 1 | 2.0 | 2.6 | 2 |
| 22 | 46 (M) | MM | Rel 2 | 2.0 | 2.9 | 4 |
| 23 | 27 (M) | ALL | CR1 | 1.8 | 3.5 | 2 |
| 24 | 52 (M) | GCT | Rel 2 | 1.8 | 11.2 | 1 |
| 25 | 32 (M) | GCT | CR3(+) | 1.8 | 5.1 | 1 |
| 26 | 32 (M) | GCT | CR3(+) | 1.8 | 60.1 | 1 |
| 27 | 26 (M) | GCT | CR2 | 1.8 | 27.6 | 1 |
| 28 | 28 (M) | GCT | CR3(+) | 1.8 | 40.8 | 1 |
| 29 | 28 (M) | GCT | Rel 1 | 1.8 | 15.2 | 1 |
| 30 | 23 (M) | GCT | CR2 | 1.8 | 6.0 | 1 |
| 31 | 34 (M) | GCT | Rel 1 | 1.8 | 6.4 | 1 |
| 32 | 26 (F) | LYM-HD | Rel 1 | 1.8 | 27.9 | 1 |
| 33 | 29 (M) | LYM-HD | CR2 | 1.8 | 2.6 | 1 |
| 34 | 52 (M) | LYM-HD | CR2 | 1.8 | 8.4 | 1 |
| 35 | 49 (M) | LYM-HD | CR2 | 1.8 | 2.4 | 1 |
| 36 | 26 (M) | LYM-NHL | CR1 | 1.8 | 5.3 | 1 |
| 37 | 44 (M) | LYM-NHL | CR3(+) | 1.8 | 4.1 | 1 |
| 38 | 55 (F) | LYM-NHL | Rel 1 | 1.8 | 2.0 | 2 |
| 39 | 70 (M) | LYM-NHL | CR2 | 1.8 | 16.0 | 1 |
| 40 | 31 (F) | LYM-NHL | CR1 | 1.8 | 2.2 | 1 |
| 41 | 43 (F) | LYM-NHL | Rel 1 | 1.8 | 2.0 | 2 |
| 42 | 47 (F) | LYM-NHL | CR3(+) | 1.8 | 1.9 | 4 |
| 43 | 16 (F) | ALL | CR1 | 1.6 | 3.1 | 3 |
| 44 | 36 (M) | GCT | CR3 (+) | 1.6 | 14.6 | 1 |
| 45 | 46 (M) | GCT | CR2 | 1.6 | 14.1 | 1 |
| 46 | 27 (M) | GCT | Rel 1 | 1.6 | 20.5 | 1 |
| 47 | 34 (M) | GCT | CR3(+) | 1.6 | 18.1 | 1 |
| 48 | 24 (M) | GCT | CR1 | 1.6 | 4.9 | 1 |
| 49 | 36 (M) | GCT | Rel 1 | 1.6 | 2.3 | 2 |
| 50 | 19 (M) | LYM-HD | Rel 1 | 1.6 | 19.9 | 1 |
| 51 | 30 (F) | LYM-HD | CR2 | 1.6 | 2.5 | 2 |
| 52 | 42 (M) | LYM-HD | Rel 2 | 1.6 | 6.5 | 1 |
| 53 | 16 (F) | LYM-HD | CR1 | 1.6 | 24.2 | 1 |
| 54 | 65 (F) | LYM-HD | CR3(+) | 1.6 | 2.4 | 1 |
| 55 | 48 (M) | LYM-NHL | CR2 | 1.6 | 14.3 | 1 |
| 56 | 44 (M) | LYM-NHL | Prim ref | 1.6 | 21.0 | 1 |
| 57 | 44 (M) | LYM-NHL | Rel 1 | 1.6 | 25.5 | 1 |
| 58 | 32 (M) | LYM-NHL | CR1 | 1.6 | 0.0 | 0 |
| 59 | 42 (M) | LYM-NHL | CR1 | 1.6 | 26.6 | 1 |
| 60 | 32 (F) | LYM-NHL | Rel 1 | 1.6 | 0.2 | 2 |
| 61 | 67 (M) | MM | Prim ref | 1.6 | 0.0 | 0 |
| 62 | 54 (F) | MM | Rel 1 | 1.6 | 3.7 | 2 |
| 63 | 57 (F) | MM | Rel 1 | 1.6 | 0.1 | 1 |

AML, acute myeloid leukaemia; ChC, choriocarcinoma; GCT, germ-cell tumour; LYM-HD, Hodgkin's disease; LYM-NHL, non Hodgkin's lymphoma; MM, myeloma; ALL, acute lymphoblastic leukaemia; CR1, first remission; CR2, second remission; Prim ref, primary refractory disease; Rel 1, first relapse; Rel 2, second relapse; CR3(+), third remission or more advanced disease.

© Cancer Research Campaign 1998

weight < 70 kg, 480 μg if ≥ 70 kg) was started on day 3 and given daily subcutaneously until leukapheresis was completed. Patients were discharged from hospital on day 3, taking prophylactic ciprofloxacin (500 mg twice daily). Toxicity of the mobilization procedure was assessed by the development of significant (WHO grade 2–4) oropharyngeal mucositis, the requirement for blood or platelet transfusions, the development of sepsis necessitating parenteral antibiotic therapy and the rehospitalization incidence.

Leukapheresis was commenced when the preceding day's circulating $CD34^+$ cell concentration predicted that an adequate collection would be obtained the following day, as previously described (Elliott et al, 1996). Assays for circulating $CD34^+$ cell concentrations were initiated from the twelfth day following etoposide administration. All collections were harvested using a Cobe Spectra (Cobe Laboratories, Quedgeley, UK), with a target processing of 2.5 times the estimated patient blood volume. If an insufficient $CD34^+$ yield resulted from the first leukapheresis further collections were obtained on subsequent days.

$CD34^+$ concentrations in both blood samples and leukapheretic products were measured flow cytometrically by dual staining for CD45 and CD34. The $CD45^+$ cell population was gated and then analysed for the percentage of $CD34^+$ cells (Sutherland et al, 1994). The $CD34^+$ concentration was derived by reference to the white cell count of the sample.

### RESULTS

### Patient characteristics

Patient characteristics are shown in Table 1. Thirty-one (49%) of the 63 patients had lymphoma [Hodgkin's disease (HD) or non-Hodgkin's lymphoma (NHL)], 24 (38%) germ cell tumour (GCT) and the remaining eight patients (13%) had myeloma ($n$ = 4), acute leukaemia ($n$ = 3) or choriocarcinoma ($n$ = 1). All patients with lymphoma had received first-line therapy with either BEMOP-CA (bleomycin, etoposide, methotrexate, vincristine, prednisolone, cyclophosphamide, doxorubicin) or CHOP (cyclophosphamide, doxorubicin, vincristine, prednisolone), and second-line therapy with MOPP (mustine, vincristine, procarbazine, prednisolone) if relapsed HD or DHAP (dexamethasone, cytosine arabinoside, cisplatinum) if relapsed NHL. All patients with GCT had received first-line therapy with POMB-ACE (cisplatinum, vincristine, methotrexate, bleomycin, actinomycin, etoposide, cyclophosphamide), and second-line therapy with an alternating cisplatinum/taxol–etoposide/taxol schedule. The patients with myeloma had received as a minimum melphalan and/or VAD therapy (vincristine, doxorubicin, dexamethasone). Forty-seven patients (75%) were male, and patient age ranged from 16 to 70 years (median 36). Fifty-five patients (87%) had disease beyond first remission and had received two or more chemotherapy regimens before the mobilization procedure. Seven patients (four with myeloma, two with lymphoma and one with acute leukaemia) had previously failed mobilization therapy with cyclophosphamide (2–4 g $m^{-2}$) and G-CSF, based on actual yields obtained and also on peak blood $CD34^+$ concentrations of less than $6 \times 10^6\ l^{-1}$ (Elliott et al, 1996).

### Toxicity

Significant oropharyngeal mucositis requiring opiate analgesia developed in four (6%) patients following etoposide. This complication was sufficiently severe in three patients to necessitate hospitalization. In total, 23 patients (37%) were readmitted to hospital during the mobilization procedure. In 20 (32%) cases this was due to sepsis requiring parenteral antibiotic therapy. Haematological toxicity requiring support with blood or platelet transfusion developed in 28 (44%) patients. There were no procedure-related mortalities observed in this study.

The incidences of these toxicities and rehospitalization appeared to be lowest in the group of patients who received 1.6 g $m^{-2}$ etoposide (Table 2). In particular, 4 of these 21 (19%) patients required readmission to hospital compared with 8 of 20 (40%) and 11 of 22 (50%) patients in the 1.8 g $m^{-2}$ and 2.0 g $m^{-2}$ groups respectively. However, only the comparison between rehospitalization incidence in the 1.6 g $m^{-2}$ and 2.0 g $m^{-2}$ groups reached statistical significance ($P$ = 0.03, two-tailed chi-square test); all other comparisons were non-significant.

### Leukaphereses and PBPC yields

The number of leukaphereses performed and the $CD34^+$ cells collected are shown in Table 1. The first day of leukapheresis in these 63 patients was at a median of 13 days following etoposide therapy (range 12–19 days), and was not different in the three groups of etoposide dosage (data not shown). Three patients (1 in the 2.0 g $m^{-2}$ and two in the 1.6 g $m^{-2}$ etoposide groups) failed to mobilize (as indicated by the peripheral blood $CD34^+$ concentration) and were not leukapheresed. A further seven patients (two each in the 2.0 g $m^{-2}$ and 1.6 g $m^{-2}$ groups, three in the 1.8 g $m^{-2}$ group) failed to achieve total yields of $2.0 \times 10^6$ $CD34^+$ cells $kg^{-1}$. Mobilization procedures that produced yields of greater than this target number were achieved in 53 (84%) patients. The median number of $CD34^+$ cells collected in all 63 patients was $5.3 \times 10^6\ kg^{-1}$, in an average of 1.5 leukaphereses (Table 3). Median yields ($\times 10^6$ $CD34^+$ cells $kg^{-1}$) in the three etoposide dosage groups were 4.7 (2.0 g $m^{-2}$), 5.7 (1.8 g $m^{-2}$) and

**Table 2** Toxicity

| Toxicity [no. of patients> (%)] | Etoposide dose (g $m^{-2}$) 2.0 ($n$ = 22) | 1.8 ($n$ = 20) | 1.6 ($n$ = 21) | Total ($n$ = 63) |
|---|---|---|---|---|
| Grade 2–4 (WHO) mucositis | 3 (14%) | 1 (5%) | 0 (0%) | 4 (6%) |
| Blood or platelet transfusion | 11 (50%) | 10 (50%) | 7 (33%) | 28 (44%) |
| Parenteral antibiotics | 10 (45%) | 6 (30%) | 4 (19%) | 20 (32%) |
| Hospitalization[a] | 11 (50%) | 8 (40%) | 4 (19%) | 23 (37%) |

[a]1.6 g $m^{-2}$ vs 2.0 g $m^{-2}$, $P$ = 0.03. All other comparisons non-significant.

 © Cancer Research Campaign 1998

**Table 3** PBPC yields

| | Etoposide dose (g $m^{-2}$) | | | |
|---|---|---|---|---|
| | **2.0** ($n$ = 22) | **1.8** ($n$ = 20) | **1.6** ($n$ = 21) | **All patients** ($n$ = 63) |
| CD34⁺ cells (median) × $10^6$ $kg^{-1}$ collected (range) | 4.7 (0.0–27.1) | 5.7 (1.9–60.1) | 6.5 (0.0–26.6) | 5.3 (0.0–60.1) |
| Number (median) of leukaphereses (range) | 2 (0–4)[a] | 1 (1–4) | 1 (0–4)[b] | 1 (0–4) |

[a]Including one patient who was not leukapheresed. [b]Including two patients who were not leukapheresed.

**Table 4** Etoposide/G-CSF mobilization in patients previously failing cyclophosphamide/G-CSF

| Diagnosis[a] | Cyclophosphamide/G-CSF (× $10^6$ CD34⁺ cells/$kg^{-1}$) | Etoposide/G-CSF (× $10^6$ CD34⁺ cells $kg^{-1}$) | Etoposide dose (g $m^{-2}$) | Number of leukaphereses |
|---|---|---|---|---|
| LYM-NHL | 0.3 | 5.8 | 2.0 | 2 |
| MM | 0.1 | 2.9 | 2.0 | 4 |
| LYM-NHL | 1.4 | 2.6 | 2.0 | 2 |
| ALL | 0.0 | 3.5 | 1.8 | 2 |
| MM | 0.1 | 0.0 | 1.6 | 0 |
| MM | 0.0 | 0.1 | 1.6 | 1 |
| MM | 1.1 | 3.7 | 1.6 | 2 |

[a]For abbreviations see Table 1.

6.5 (1.6 g $m^{-2}$), and the average number of leukaphereses required were 1.7 (2.0 g $m^{-2}$), 1.3 (1.8 g $m^{-2}$) and 1.5 (1.6 g $m^{-2}$).

Five of the seven patients who had previously failed cyclophosphamide with G-CSF mobilization achieved the target yield following etoposide/G-CSF (Table 4). The two patients in this group who did not mobilize successfully following etoposide/G-CSF had an underlying diagnosis of myeloma and both received 1.6 g $m^{-2}$ etoposide.

### DISCUSSION

Several studies have suggested that the use of PBPCs to support high-dose therapy has advantages compared with autologous bone marrow (Elias et al. 1992; To et al. 1992; Chao et al. 1993; Schmitz et al. 1996). These benefits may include shorter median duration of neutropenia and thrombocytopenia, fewer febrile episodes with reduced antibiotic requirement, shorter hospitalization and lower procedural costs.

The procurement of PBPCs for this purpose has most often been accomplished using a combination of cyclophosphamide with G-CSF, but not all patients mobilize successfully. In particular, leukapheretic yields may be suboptimal in patients who have received several preceding regimens of chemotherapy (Haas et al. 1994; Dreger et al. 1995). Strategies employed to improve the results of mobilization have included increasing the dose of cyclophosphamide administered or using combinations of cytotoxic drugs (Lie et al. 1996; Demirer et al. 1997; McQuaker et al. 1997). The majority of patients (87%) in the present study had received at least two prior schedules of chemotherapy, and the mobilization therapy was successful in 53 of 63 patients (84%). In addition, five of seven patients who had previously failed to achieve a target collection of 2.0 × $10^6$ CD34⁺ cells $kg^{-1}$ with cyclophosphamide/G-CSF mobilization yielded an adequate harvest following etoposide/G-CSF. These data confirm that high-dose etoposide with G-CSF is an effective mobilization regimen in the majority of patients, despite substantial previous therapy or an initial failure to mobilize PBPCs with cyclophosphamide/G-CSF.

The percentage of patients who mobilized PBPCs adequately and the median number of CD34⁺ cells $kg^{-1}$ obtained were not different between the three groups of etoposide dosage. In contrast, the toxicity experienced was lowest in those who received 1.6 g $m^{-2}$, with only 4 of these 21 patients needing readmission to hospital. In general, however, the toxic complications and rehospitalization frequency appear to have been greater than in the two previous reports of high-dose etoposide with G-CSF/GM-CSF. In the first study none of the patients evaluated had been heavily pretreated (Gianni et al. 1992), and in the second approximately 50% of the patients studied had breast cancer (Copelan et al. 1997). In a separate study at this centre PBPCs have been collected from more than 50 patients with high-risk primary breast cancer following mobilization with combination chemotherapy and G-CSF, and in none of these has readmission to hospital been required, suggesting that both previous therapy and underlying diagnosis may impact on procedural complications.

The equivalent cell yields in the three groups of etoposide dosage may suggest that a further reduction might eliminate toxicity without impairing PBPC mobilization. However, although disease response was not specifically evaluated in this study, there is evidence that high-dose etoposide is an effective agent in a variety of tumours (Postmus et al. 1984; Marangolo et al. 1989; Herzig, 1991; Bezwoda et al. 1992). It may therefore be advantageous to employ an etoposide dose sufficient to exploit this potential anti-tumour activity. These data indicate that 1.6 g $m^{-2}$ may represent a reasonable compromise for this purpose, enabling the successful mobilization of adequate PBPCs for the support of subsequent high-dose therapy but with acceptable toxicity.

© Cancer Research Campaign 1998 

### REFERENCES

Bezwoda WR. Seymour L and Ariad S (1992) High-dose etoposide in treatment of metastatic breast cancer. *Oncology* **49**: 104–107

Chao NJ. Schriber JR. Grimes K. Long GD. Negrin RS. Raimondi CM. Horning SJ. Brown SL. Miller L and Blume KG (1993) Granulocyte colony-stimulating factor 'mobilized' peripheral blood progenitor cells accelerate granulocyte and platelet recovery after high-dose chemotherapy. *Blood* **81**: 2031–2035

Copelan EA. Ceselski SK. Ezzone SA. Lasky LC. Penza SL. Bechtel TP. Klein JL. Hehmeyer DM. Scholl MD. Marshall DD. Elder PJ. Risley GL and Avalos BR (1997) Mobilization of peripheral-blood progenitor cells with high-dose etoposide and granulocyte colony-stimulating factor in patients with breast cancer, non-Hodgkin's lymphoma, and Hodgkin's disease. *J Clin Oncol* **15**: 759–765

Demirer T. Buckner CD. Storer B. Lilleby K. Rowley S. Clift R. Appelbaum FR. Storb R and Bensinger WI (1997) Effect of different chemotherapy regimens on peripheral-blood stem-cell collections in patients with breast cancer receiving granulocyte colony-stimulating factor. *J Clin Oncol* **15**: 684–690

Diaz Mediavilla J. Llorente L. Martinez R. Alvarez Carmona A. Jorda J. Del Potro E. Gonzalez A. Morales D. Asenjo S. Farinas M. Saez I and Villegas A (1996) Autotransplantation of peripheral blood stem cells mobilized by G-CSF in hematological malignancies: evidence for rapid and long-term sustained hematopoietic reconstitution. *Leuk Lymphoma* **20**: 327–332

Dreger P. Kloss M. Petersen B. Haferlach T. Loffler H. Loeffler M and Schmitz N (1995) Autologous progenitor cell transplantation: prior exposure to stem cell-toxic drugs determines yield and engraftment of peripheral blood progenitor cell but not of bone marrow grafts. *Blood* **86**: 3970–3978

Elias AD. Ayash L. Anderson KC. Hunt M. Wheeler C. Schwartz G. Tepler I. Mazanet R. Lynch C and Pap S (1992) Mobilization of peripheral blood progenitor cells by chemotherapy and granulocyte-macrophage colony-stimulating factor for hematologic support after high-dose intensification for breast cancer. *Blood* **79**: 3036–3044

Elliott C. Samson DM. Armitage S. Lyttelton MP. McGuigan D. Hargreaves R. Giles C. Abrahamson G. Abboudi Z. Brennan M and Kanfer EJ (1996) When to harvest peripheral-blood stem cells after mobilization therapy: Prediction of CD34-positive cell yield by preceding day CD34-positive concentration in peripheral blood. *J Clin Oncol* **14**: 970–973

Gianni AM. Siena S. Bregni M. Tarella C. Stern AC. Pileri A and Bonadonna G (1989) Granulocyte-macrophage colony-stimulating factor to harvest circulating haemopoietic stem cells for autotransplantation. *Lancet* **2**: 580–585

Gianni AM. Bregni M. Siena S. Magni M. Di Nicola M. Lombardi F. Tarella C. Pileri A and Bonadonna G (1992) Granulocyte–macrophage colony-stimulating factor or granulocyte colony-stimulating factor infusion makes high-dose etoposide a safe outpatient regimen that is effective in lymphoma and myeloma patients. *J Clin Oncol* **10**: 1955–1962

Haas R. Mohle R. Fruhauf S. Goldschmidt H. Witt B. Flentje M. Wannenmacher M and Hunstein W (1994) Patient characteristics associated with successful mobilizing and autografting of peripheral blood progenitor cells in malignant lymphoma. *Blood* **83**: 3787–3794

Herzig RH (1991) High-dose etoposide and marrow transplantation. *Cancer* **67**: 292–298

Lie AK. Rawling TP. Bayly JL and To LB (1996) Progenitor cell yield in sequential blood stem cell mobilization in the same patients: insights into chemotherapy dose escalation and combination of haemopoietic growth factor and chemotherapy. *Br J Haematol* **95**: 39–44

McQuaker IG. Haynes AP. Stainer C. Anderson S and Russell NH (1997) Stem cell mobilization in resistant or relapsed lymphoma: superior yield of progenitor cells following a salvage regimen comprising ifosphamide, etoposide and epirubicin compared to intermediate-dose cyclophosphamide. *Br J Haematol* **98**: 228–233

Marangolo M. Rosti G. Amadori D. Leoni M. Ardizzone A. Fiorentini G. Cruciani G. Tienghi A. Ravaioli A. Sebastiani L. Turci D. Cotignoli T. Argnani M and Flamini E and Rosso R (1989) High-dose etoposide and autologous bone marrow transplantation as intensification treatment in small cell lung cancer: a pilot study. *Bone Marrow Transplant* **4**: 405–408

Mross K. Bewermeier P. Kruger W. Stockschlader M. Zander A and Hossfeld DK (1994) Pharmacokinetics of undiluted or diluted high-dose etoposide with or without busulfan administered to patients with hematologic malignancies. *J Clin Oncol* **12**: 1468–1474

Pettengell R. Testa NG. Swindell R. Crowther D and Dexter TM (1993) Transplantation potential of hematopoietic cells released into the circulation during routine chemotherapy for non-Hodgkin's lymphoma. *Blood* **82**: 2239–2248

Postmus PE. Mulder NH. Sleijfer DT. Meinesz AF. Vriesendorp R and de Vries EG (1984) High-dose etoposide for refractory malignancies: a phase I study. *Cancer Treat Rep* **68**: 1471–1474

Richman CM. Weiner RS and Yankee RA (1976) Increase in circulating stem cells following chemotherapy in man. *Blood* **47**: 1031–1039

Rosenfeld CS. Bolwell B. LeFever A. Taylor R. List A. Fay J. Collins R. Andrews F. Pallansch P. Schuster MW. Resta D. Levitt D and Nemunaitis J (1996) Comparison of four cytokine regimens for mobilization of peripheral blood stem cells: IL-3 alone and combined with GM-CSF or G-CSF. *Bone Marrow Transplant* **17**: 179–183

Schmitz N. Linch DC. Dreger P. Goldstone AH. Boogaerts MA. Ferrant A. Demuynck HM. Link H. Zander A and Barge A (1996) Randomised trial of filgrastim-mobilised peripheral blood progenitor cell transplantation versus autologous bone-marrow transplantation in lymphoma patients. *Lancet* **347**: 353–357

Schwartzberg LS. Birch R. Hazelton B. Tauer KW. Lee Jr P. Altemose R. George C. Blanco R. Wittlin F and Cohen J (1992) Peripheral blood stem cell mobilization by chemotherapy with and without recombinant human granulocyte colony-stimulating factor. *J Hematother* **1**: 317–327

Sutherland DR. Keating A. Nayar R. Anania S and Stewart AK (1994) Sensitive detection and enumeration of $CD34^{+}$ cells in peripheral and cord blood by flow cytometry. *Exp Hematol* **22**: 1003–1010

To LB. Shepperd KM. Haylock DN. Dyson PG. Charles P. Thorp DL. Dale BM. Dart GW. Roberts MM and Sage RE (1990) Single high doses of cyclophosphamide enable the collection of high numbers of hemopoietic stem cells from the peripheral blood. *Exp Hematol* **18**: 442–447

To LB. Roberts MM. Haylock DN. Dyson PG. Branford AL. Thorp D. Ho JQ. Dart GW. Horvath N. Davy ML. Olweny CLM. Abdi E and Juttner CA (1992) Comparison of haematological recovery times and supportive care requirements of autologous recovery phase peripheral blood stem cell transplants, autologous bone marrow transplants and allogeneic bone marrow transplants. *Bone Marrow Transplant* **9**: 277–284

 © Cancer Research Campaign 1998